# Community-based causal evidence that high habitual caffeine consumption alters distinct polysomnography-derived sleep variables

**DOI:** 10.1177/02698811251368364

**Published:** 2025-10-18

**Authors:** Benjamin Stucky, Leonard Henckel, Marloes H. Maathuis, José Haba-Rubio, Pedro Marques-Vidal, Francesca Siclari, Raphaël Heinzer, Hans-Peter Landolt

**Affiliations:** 1 Institute of Pharmacology and Toxicology, University of Zurich, Switzerland; 2 School of Mathematics and Statistics, University College Dublin, Ireland; 3 Independent Researcher, The Netherlands; 4 Center for Investigation and Research in Sleep, Lausanne University Hospital, Switzerland; 5 Department of Medicine, Internal Medicine, Lausanne University Hospital and University of Lausanne, Switzerland; 6 The Netherlands Institute for Neuroscience, Amsterdam, The Netherlands; 7 The Sense Innovation and Research Center, Lausanne and Sion, Switzerland; 8 Pulmonary Department, Lausanne University Hospital, Switzerland; 9 Sleep and Health Zurich, University Center of Competence, University of Zurich, Switzerland

**Keywords:** habitual caffeine consumption, sleep duration, sleep depth, Mendelian randomization, EEG, public health

## Abstract

**Background:**

Controlled laboratory studies demonstrate that caffeine acutely impairs sleep quality. However, the impact of daily caffeine intake, which is common in society, on community-derived physiological sleep measures is unknown.

**Aims:**

Because good quality sleep is important for general health and well-being, we explored causal effects of habitual caffeine consumption on objective and subjective sleep variables collected at home.

**Methods:**

We used dedicated, two-sample Mendelian Randomization (MR) and causal matching methods, including MR-Egger, inverse variance weighting, and weighted median, to analyze large community-based datasets taken from the UK Biobank (*n* = 485,511) and the HypnoLaus (*n* = 1702) cohorts.

**Results:**

While self-rated sleep quality and morningness–eveningness did not differ, all statistical models revealed that four or more caffeinated beverages per day shorten total sleep time when compared to fewer caffeine containing drinks per day. The estimated reductions in sleep length varied from 11 to 229 minutes. Intriguingly, consistent with the homeostatic facet of sleep-wake regulation, the shorter sleep in high habitual caffeine consumers was characterized by increased non-rapid-eye movement sleep depth as measured by all-night electrical brain activity.

**Conclusions:**

The data show that high habitual caffeine intake alters the characteristics of sleep in the general population, while sparing the major physiological principles of sleep-wake regulation possibly due to adaptation.

## Introduction

Caffeine use in humans has a rich and long history (Fredholm, 2011). Today, caffeine is considered the most widely consumed psychoactive substance in the world (Fredholm et al., 1999). It occurs naturally and is readily available in common foods and beverages, including coffee, tea, energy drinks, and chocolate (Heckman et al., 2010). Wastewater analyses estimate that people in Europe consume about 86–263 mg of caffeine per day, which equals roughly 1–3 cups of coffee (Gracia-Lor et al., 2017). Caffeine intake is motivated by its ability to promote alertness, enhance taste and smell, foster social interactions, increase energy, and manage stress (Ágoston et al., 2018; Mahoney et al., 2019).

The prevalent use of caffeine raises questions regarding its potential health consequences. A key aspect of caffeine-related health is sleep quality (Luyster et al., 2012). Undisturbed sleep is required for many physical and cerebral processes, including cardiovascular, immune, and memory processes, as well as other functions like emotion regulation (Besedovsky et al., 2012; Mendelsohn and Larrick, 2013; Palmer and Alfano, 2017; Siegel, 2005; Walker and Stickgold, 2006). Carefully controlled polysomnographic recordings including electroencephalographic (EEG) measurements revealed that acute caffeine intake prolongs the time to fall sleep and wakefulness after sleep onset, and reduces sleep efficiency, sleep duration, and non-rapid-eye-movement (NREM) sleep intensity (Clark and Landolt, 2017; Drake et al., 2013; Gardiner et al., 2023). However, such studies typically enroll habitual caffeine consumers and instruct them to abstain prior to the experiment. Hence, they investigate the effects of caffeine after abstinence, which may not reflect potential adaptations to chronic intake (Weibel et al., 2020), which is common in society (Martyn et al., 2018).

Very little is currently known about the effects of chronic caffeine intake on sleep. Whereas early data in cats indicated that 21 days of chronic caffeine administration lead to more superficial sleep and difficulties falling asleep (Sinton and Petitjean, 1989), some recent evidence suggests that repeated caffeine intake increases NREM sleep intensity. More specifically, a study in mice revealed that 2 weeks of continuous caffeine consumption enhanced both the amplitude of the daily sleep-wake cycle, as well as behavioral and EEG markers of sleep intensity (Aframian et al., 2023; Panagiotou et al., 2019). Additional effects seen in mice included increased mean cerebral blood volume and hear rate variability during rest phases (Aframian et al., 2023). In healthy men, chronic caffeine intake over 10 consecutive days did not reduce NREM sleep intensity but delayed the occurrence of REM sleep (Weibel et al., 2020, 2021). These findings suggest that chronic caffeine consumption may induce tolerance to the acute effects on sleep mentioned above (Evans and Griffiths, 1999; Holtzman et al., 1991; Juliano and Griffiths, 2004; Reichert et al., 2022). Nonetheless, controlled laboratory protocols have limitations because they do not reflect the long-term habits seen in the general population. In addition, the artificial laboratory setting does not allow the participants to maintain their regular sleep routines.

A promising approach to address these limitations and to assess causal effects in epidemiological datasets is the use of statistical tools such as Mendelian Randomization (MR; Davey Smith and Ebrahim, 2003; Emdin et al., 2017; Lawlor et al., 2008; Smith and Ebrahim, 2004). These methods use genetic variants linked to a behavior, like caffeine use, to estimate causal effects on a variable of interest, like sleep quality. Habitual caffeine intake is modulated by genetic influences (Cornelis et al., 2007, 2015, 2016; Loftfield et al., 2018; Yang et al., 2010) and thus lends itself to MR methodologies. Previous MR research on the relationships between habitual caffeine intake and sleep-associated outcomes, only relied on subjective sleep estimates (Deng et al., 2023; Treur et al., 2018), which can be systematically biased and often deviate from objective measures (Bianchi et al., 2013; Hermans et al., 2020; Lauderdale et al., 2008).

By contrast, causal effects of habitual caffeine intake on objective markers of sleep quality are unknown. To start filling this knowledge gap, we estimated the causal effects of habitual caffeine consumption on objective and subjective sleep variables recorded at home, using three distinct two-sample MR methods in the two large cohorts, UK Biobank (Sudlow et al., 2015) and HypnoLaus (Haba-Rubio et al., 2015; Heinzer et al., 2015).

## Materials and methods

### Study population

This study was conducted using the UK Biobank and HypnoLaus cohorts. The UK Biobank is a very large open access prospective study conducted in the UK (Sudlow et al., 2015). We used the subpopulation that contains information on caffeine intake and genetic information, amounting to a total of 485,511 participants. The UK Biobank was used to estimate the association of various gene variants with caffeine intake. The selection process is described below. The HypnoLaus cohort, which belongs to the CoLaus/PsyCoLaus cohort, is a population-based study of Lausanne, Switzerland (Haba-Rubio et al., 2015; Heinzer et al., 2015). The HypnoLaus dataset contains caffeine intake, genetic, and objective sleep data collected from at-home polysomnography in 1755 participants. The HypnoLaus cohort was used to estimate the association of each gene variant with several objective and subjective measure of sleep quality.

### Caffeinated beverages per day

**Figure 1.**
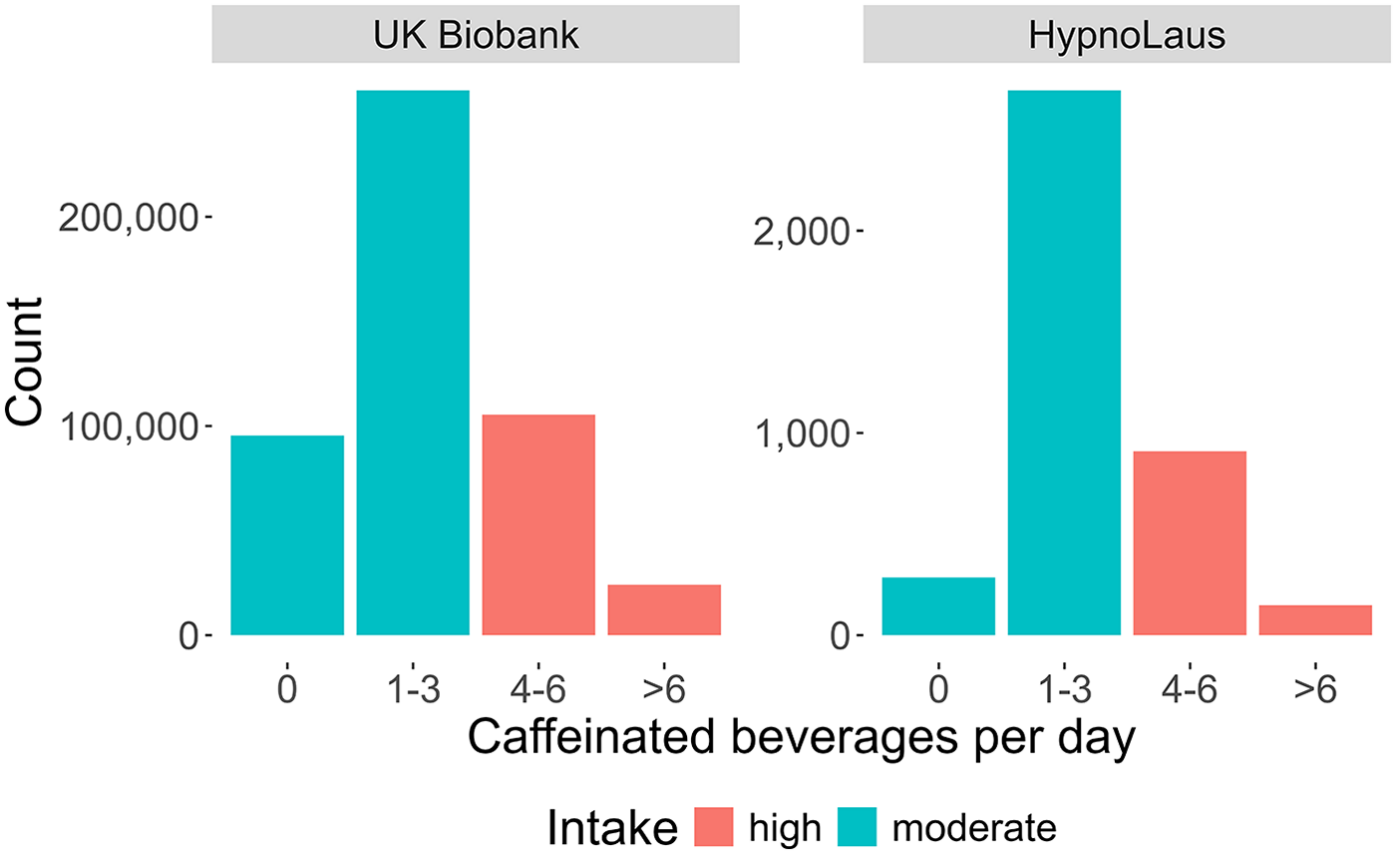
Distributions of self-reported intake of caffeinated beverages per day. Distributions are shown for participants of the UK Biobank (*n* = 485,511) and HypnoLaus (*n* = 1702) cohorts. The different colors highlight the high (red; ⩾4 caffeinated beverages per day) and moderate (cyan; ⩽3 caffeinated beverages per day) intake groups. Please note the different scales (*y*-axes) between the two datasets.

The variable caffeinated beverage per day was measured in the HypnoLaus dataset with granularity: 0 cups/day, 1–3 cups/day, 4–6 cups/day, >6 cups/day. The UK Biobank contained more detailed information on caffeine intake at a higher granularity, for example, different beverage types (tea, coffee, caffeinated sodas, etc.) were listed. For comparability, we combined the intake of these various beverage types and reduced the intake detail to match the cups of caffeinated beverage intake categories from the HypnoLaus dataset. The resulting distributions are shown in Figure 1. To streamline the MR analyses and obtain more balanced caffeine intake groups, we split the intake habits into high (⩾4 caffeinated drinks/day) and moderate (⩽3 caffeinated drinks/day) caffeine use. This approach helps balance the data but precludes a direct comparison between people who habitually consume any caffeine and those who do not.

### Sleep variables in the HypnoLaus dataset

Participants performed a full night polysomnography at home (Titanium, Embla^®^ Flaga, Reykjavik, Iceland). Sleep recordings followed standard guidelines from the American Academy of Sleep Medicine (AASM) 2007 (Iber, 2007) and included: EEG leads (F3, F4, C1, C2, O1, and O2, 256 Hz sampling rate); electrooculography (left and right); electromyography (chin and anterior tibialis muscle); electrocardiography (one lead); oxygen saturation; airflow (nasal cannula); abdominal and thoracic respiratory efforts; snoring; and body position. Sleep data were visually scored according to the AASM guidelines 2007 (Iber, 2007).

We considered the following six objective measure of sleep quality: Total sleep time, sleep latency (time between lights-off to N2 sleep), number of awakenings, percentage of REM sleep per total sleep time, and EEG delta (1–4 Hz) and sigma (12–16 Hz) power in NREM sleep relative to the total power between 0.5 and 30 Hz. We chose these distinct objective sleep measures, to capture different aspects of the complex and multifaceted quality of sleep. For a detailed explanation of the EEG data preprocessing and artifact handling, please see Lecci et al. (2020).

To complement our analyses and verify previous studies on subjective sleep measures (Treur et al., 2018), we also included the following validated sleep quality questionnaires: Pittsburgh Sleep Quality Index (PSQI, global score; Buysse et al., 1989), Epworth Sleepiness Scale (ESS; Johns, 1991), and Morningness-Eveningness Questionnaire (MEQ; Horne and Ostberg, 1976). The PSQI is the most commonly used self-perceived sleep quality questionnaire, the ESS captures daytime sleepiness, and the MEQ assesses the preference for being active in the morning or evening, so-called diurnal preference.

### Mendelian randomization

Instrumental variable estimators can estimate causal effects of a treatment on an outcome even in the presence of unmeasured confounding or reverse causality, that is, the outcome causing the treatment (Angrist et al., 1996; Basmann, 1957). In general, they require an auxiliary variable, referred to as instrument, which has to be (i) robustly associated with the treatment; (ii) not share common causes with the outcome; and (iii) affect the outcome only via the treatment. While precondition (i) can be verified statistically, ensuring preconditions (ii) and (iii) requires subject matter knowledge (Glymour et al., 2012; Lawlor et al., 2008). MR is a special case of an instrumental variable estimator, where a gene variant is used as an instrument (Lawlor et al., 2008). Gene variants (i.e., single-nucleotide polymorphisms (SNPs)) are natural instruments because they cannot be affected by most observational covariates. As a result, precondition (ii) mostly holds. Precondition (iii), which is often referred to as the no-pleiotropy assumption, can still be violated, thus requiring careful selection of SNPs. For an overview of these assumptions in an MR context, please see Didelez and Sheehan (2007). To further protect against violations of the no-pleiotropy assumption, newer MR methods typically use multiple instruments (Bowden et al., 2015, 2016; Guo et al., 2018). We used three such estimators. First, the MR-Egger estimator, which is consistent under the instrument strength independent of direct effect (InSIDE) assumption. That is, the association between the outcome and the instruments is independent of the association between the treatment and the instruments. In principle, InSIDE may hold even if all SNPs are invalid. Second, the inverse variance weighted (IVW) average of Wald ratios (Lawlor et al., 2008). This estimator is consistent if all individual SNPs are valid. The IVW average has the major advantage that it is more statistically accurate than any of the individual Wald ratios, which tend to suffer from low accuracy (Kinal, 1980). And third, the weighted median of Wald ratios (Bowden et al., 2016). This estimator is consistent if more than half of the weight comes from valid instruments.

We used a two-sample MR approach. This procedure can have some advantages compared to one-sample MR. It can increase statistical power to detect effects, reduce bias due to weak instruments, and prevent underestimation of causal effects due to the winner’s curse (Lawlor, 2016). Two-sample MR methods assume that the two study populations are comparable (Lawlor, 2016).

### Causal matching

In addition to MR, we used causal matching to estimate the causal effects of habitual caffeine consumption on objective and subjective measures of sleep quality. In a randomized trial, participants are randomly assigned to groups to make sure other factors are evenly distributed. This approach is ideal to assess causal effects. In an observational setting, however, people with high habitual caffeine intake likely differ in other aspects from people with moderate caffeine intake. One way to mimic randomization is to match each high-caffeine user with a low-caffeine user having similar characteristics (Stuart, 2010). This approach might not work well if important factors affecting both caffeine use and sleep haven’t been measured, or if sleep also influences caffeine use. Because both are likely the case here, for example, due to unmeasured inter-individual differences in circadian rhythms or lifestyle factors, matching was used to corroborate the MR results.

We used Matching Frontier version 4.1.0 (King et al., 2017), which simultaneously optimizes the similarity between the groups of interest and the matched sample size. All observations of the moderate group were matched. To obtain good balance, we retained 50% of those best matches. The exact percentage of retained matches did not strongly affect the results (Supplemental Figure S1). For total sleep time, latency to N2 sleep, number of awakenings, and percentage of REM sleep per total sleep time, 609 pairs remained. For relative EEG delta (1–4 Hz) and sigma (12–16 Hz) power in NREM sleep, there were 588 pairs. The subjective variables retained 1189 pairs for PSQI, 1249 for ESS, and 1211 for the MEQ.

We used various covariates available in the HypnoLaus cohort. They included physical health (subjective overall health rating, total weekly energy expenditure excluding sleep, alcohol units per week, smoking cigarettes equivalent per day, body mass index); socio-demographic status (age, gender, self-reported ethnicity, highest education level, marital status); as well as economic characteristics (occupational position).

### SNP selection

Based on a literature search of prior publications investigating the association of genetic variants on caffeine consumption, we preselected 83 SNPs. The references and selected SNPs can be found in Supplemental Table S3. In the UK Biobank data, we calculated the *t*-value of each SNP association with caffeine intake. Due to missing SNP information, we could not calculate the *t*-values for six of the preselected SNPs. To mitigate power loss due to weak instruments, an absolute *t*-value > 8 was used as inclusion criterion for the MR analyses. This value corresponds to a slightly stricter cut-off value than the common 5 × 10^−8^ genome-wide significance p-value threshold (Fadista et al., 2016). This resulted in 30 SNPs selected for the analyses (Table 1).

**Table 1.** Selection of single-nucleotide polymorphisms.

t-value	SNP-ID	Gene(s)	Chr	MR-Egger	IVW	Median	Link	Validity
34.3	rs2472297	*CYP1A1, CYP1A2*	15			Yes	1	Implausible
32.0	rs2470893	*CYP1A1, CYP1A2*	15	Yes	Yes	Yes	1	**Plausible**
31.3	rs35107470	*AC012435.2, ARID3B*	15		Yes	Yes	1	**Plausible**
30.2	rs4410790	*AHR, AC073332.1*	7	Yes	Yes	Yes	2	**Plausible**
30.1	rs6968865	*AHR, AC073332.1*	7		Yes	Yes	2	**Plausible**
29.7	rs2472304	*CYP1A2*	15		Yes	Yes	1	**Plausible**
29.0	rs12909047	*AC012435.2, AC012435.1, UBL7-AS1*	15		Yes	Yes	1	**Plausible**
26.0	rs6968554	*AHR, AC073332.1*	7		Yes	Yes	2	**Plausible**
20.6	rs1992145	*SEMA7A*	15		Yes	Yes	1	**Plausible**
19.7	rs62005807	*CLK3*	15		Yes	Yes	1	**Plausible**
−19.2	rs10275488	*AHR, AC073332.1*	7		Yes	Yes	2	**Plausible**
18.5	rs2892838	*AHR, AC073332.1*	7		Yes	Yes	2	**Plausible**
16.0	rs762551	*CYP1A2*	15			Yes	1	Implausible
13.9	rs56113850	*CYP2A6, AC008537.1*	19	Yes		Yes	1	Implausible
11.9	rs4822492	*ADORA2A-AS1*	22				3	Implausible
11.8	rs7800944	*MLXIPL*	7	Yes		Yes		Rather implausible
11.6	rs2298383	*ADORA2A*	22				3	Implausible
11.1	rs17685	*POR*	7	Yes	Yes	Yes		**Plausible**
11.1	rs10516471	*PPP3CA*	4	Yes		Yes		Rather implausible
−11.0	rs7605062	*POTEI*	2	Yes	Yes	Yes		**Plausible**
10.1	rs5751876	*ADORA2A*	22				3	Implausible
9.9	rs1800498	*DRD2*	11	Yes		Yes	4	Implausible
9.4	rs2668822	*—*	2	Yes		Yes		Not known
8.7	rs767778	*—*	13	Yes		Yes		Not known
-8.6	rs10007278	*ARHGEF38*	4	Yes	Yes	Yes		**Plausible**
8.4	rs6575353	*PRIMA1*	14	Yes		Yes		Rather implausible
8.3	rs347306	*NOS1AP*	1	Yes		Yes		Rather implausible
8.2	rs6279	*DRD2*	11			Yes	4	Implausible
8.1	rs1571536	*GADD45G*	9	Yes		Yes		Rather implausible
8.1	rs66500423	*NUMBL*	19	Yes		Yes		Rather implausible

The *t*-value, the SNP-ID (rs-number), the associated gene(s), the chromosome number (Chr), and the MR sets for MR-Egger, inverse variance weighting (IVW), and weighted median are reported. Link: SNPs in high linkage (D’ > 0.5). Numbers indicate high linkage groups. Validity (right column) indicates whether the respective SNPs are valid instruments for MR (i.e., likely affect sleep quality only by directly modulating habitual caffeine consumption). Plausible SNPs are highlighted in the column Validity in bold.

To select SNP sets likely fulfilling the model assumptions, we first checked whether each of the 30 individual SNPs is plausibly a valid instrument. Because validity cannot be verified with statistical tests, we performed a comprehensive literature search on the functions of each SNP and whether it may affect sleep directly or indirectly, that is, not mediated by its effect on caffeine. The genes’ functionalities were either identified in papers found on Google Scholar or through the extensive gene database GeneCards (Safran et al., 2010) and the protein database UniProt (The UniProt Consortium, 2015, 2017). The associations of SNPs with sleep-related topics were either identified in papers found on Google Scholar or through the National Human Genome Research Institute (NHGRI) - European Bioinformatics Institute (EBI) genome-wide association studies Catalog (Buniello et al., 2019) and human genetic wiki SNPedia (Cariaso and Lennon, 2012). This knowledge base was the basis for constructing the following validity categories: plausible, rather plausible, unknown, rather implausible and implausible. This categorization attempts to screen potentially non-valid SNPs (see Supplemental Table S3 for further information).

Furthermore, certain combinations of alleles at different genetic locations can be inherited more frequently than chance. This effect is called Linkage Disequilibrium (LD; Reich et al., 2001; Sved and Hill, 2018). Thus, certain SNPs can be biologically connected to each other, which affects whether they are likely to fulfill the InSIDE assumption of the MR-Egger method. One way to measure LD while retaining comparability between different pairs of alleles, is the relative LD measure D prime (D’; 1964). It measures in percentage how dependent two SNPs are. We chose D’ instead of *r*^2^, another measure of LD, as D’ also captures non-linear dependencies (Smith, 2020; Supplemental Figure S2). We computed D’ for each SNP pair within a given chromosome with the online tool LDmatrix from LDlink (Machiela and Chanock, 2015) based on European populations, which included subpopulations from Great Britain and central Europe, corresponding to the locations of the cohorts used. We considered a D’ value of more than 0.5 to show high linkage as it is the halfway point of the scale.

For the MR-Egger method, we removed the variables related to adenosine receptors (Reichert et al., 2022), as these influence sleep most directly. As discussed above, MR-Egger requires a set of variables that are biologically relatively independent of each other, to fulfill the InSIDE assumption. Thus, we selected the first (based on the absolute t-value ordering) SNP member of each high LD group (D’ > 0.5), whenever there was a LD grouping present, or else single independent SNPs (Table 1 and Supplemental Figure S3). The SNP set used for IVW consisted of the plausibly valid SNPs (Table 1). For the median method, which has the least stringent requirements for SNP selection, we also removed the adenosine receptor variants due to their known direct effect on sleep (Table 1).

### Statistical analyses

We performed all analyses in *R*. First, we estimated the association between the SNPs and the self-reported average of caffeinated beverages per day with linear regression, adjusting for age and sex using the UK Biobank dataset. We did so because age and sex are independent of the SNPs considered, but predictive of the sleep outcome variables. Adjusting for these two covariates cannot render a valid SNP invalid, but may improve the accuracy of our estimates (Henckel et al., 2024; Vansteelandt and Didelez, 2018).

Second, we estimated the association between the SNPs and the six objective and three subjective measures of sleep quality considered, with linear regression, again adjusting for age and sex using the HypnoLaus dataset. We log-transformed the outcome variables sleep latency (min), number of awakenings, the PSQI global score, and the ESS score. All these variables are strictly positive with a right-skewed distribution. Furthermore, we logit-transformed NREM sigma power (%) because it displayed a heavily left-skewed distribution.

We computed Wald ratio estimates for the causal effect of high habitual caffeine consumption on the six objective and three subjective measures of sleep quality introduced above (Figure 3). To reduce the likelihood that our estimates are affected by pleiotropy, we applied three methods that combined Wald ratios to obtain a more stable estimate: MR-Egger (Bowden et al., 2015), IVW (Lawlor et al., 2008), and weighted median (Bowden et al., 2016). We applied each of the three methods using selected subsets of the available SNPs (see Table 1) with the MendelianRandomization package version 0.9.0 (Burgess and Yavorska, 2024) in *R*. We used the same method-specific SNP sets for all nine outcome variables considered.

As a baseline effect, we also computed the observational effect of habitual caffeine consumption on the nine respective outcome variables with linear regression adjusting for age and sex in the HypnoLaus dataset. To reinforce the validity of our findings, we included age as a control outcome. Age is known to not be causally influenced by caffeine intake (i.e., drinking more caffeine does not change one’s age). Thus, we expect the causal models to be non-significant for this variable, whereas the observational effect likely displays a correlation, as older participants tend to consume less caffeine.

The ethics committee/IRB of North West – Haydock Research Ethics Committee gave ethical approval for reusing the UK Biobank (REC reference: 21/NW/0157). This research has been conducted using the UK Biobank Resource under Application Number 52390. The ethics committee/IRB of the Vaud Canton (CER-VD) gave ethical approval for the HypnoLaus and CoLaus|PsyCoLaus project (CER-VD nr PB_2018-00038 (239/09))

## Results

The demographic characteristics of the study participants are summarized in Table 2 (see also Supplemental Table S1). In the UK Biobank, the intake groups differed in age (high–moderate = −0.2 years, *p* < 0.001), percentage of females (−5.8%, *p* < 0.001), BMI (+0.4 kg/m^2^, *p* < 0.001), subjective total sleep time (<0.1 hour, *p* < 0.001), and cigarettes per day in smokers (+1.7, *p* < 0.001). In the HypnoLaus cohort, the groups differed in age (−2.2 years, *p* < 0.001), polysomnography-derived total sleep time (−0.2 hour, *p* = 0.002), percentage of smokers (+11.6%, *p* < 0.001), and cigarettes per day (+3.8, *p* < 0.001). The distributions of self-reported, habitual caffeine intake in the UK Biobank and HypnoLaus cohorts were comparable, with a majority of participants reporting habitual intake of 1–3 caffeinated beverages per day (Figure 1). In the UK Biobank cohort, 19.7% reported no caffeine intake, 53.7% intake of 1–3 cups/day, 21.7% intake of 4–6 cups/day, and 5% intake of more than 6 cups/day. The respective values in the HypnoLaus cohort equaled of 7.1% consuming 0 cups/day, 66.7% consuming 1–3 cups/day, 22.5% consuming 4–6 cups/day, and 3.7% consuming more than 6 cups/day.

**Table 2.** Demographics.

*p*-value	Caffeine	*n*	Age (years)	Female (%)	BMI (kg/m^2^)	TST (h)	Smoking (%)			Cigarettes	Alcohol intake (%)				MEQ score	PSQI score	ESS score
							No	Yes	Former	Per day	daily	Weekly	Less	Never			
	UK Biobank cohort																
	Moderate	355,914	56.6 ± 8.1	55.8	27.3 ± 4.8	7.1 ± 1.3	56.6	8.9	34.1	14.7 ± 8.5	19.9	48.7	22.9	8.5	—	—	—
	High	129,597	56.4 ± 8	50	27.7 ± 4.8	7.1 ± 1.2	48.8	14.8	36.1	16.4 ± 8.5	21.8	49.5	21.9	6.7	—	—	—
*p*	—	—	<0.001	<0.001	<0.001	<0.001	0.721			<0.001	0.368				—	—	—
	HypnoLaus cohort																
	Moderate	1262	59.1 ± 10.6	52.3	26.2 ± 4.4	6.7 ± 1.2	45.6	15.1	39.1	13.4 ± 10	25.9	42.5	17.5	0.2	53.3 ± 4.2	5.1 ± 3.3	6 ± 3.7
	High	464	56.9 ± 10.3	48.7	26.3 ± 4.5	6.5 ± 1.2	31.3	26.7	41.8	17.2 ± 10.7	18.8	50.2	17.7	0.7	53.2 ± 4.4	5.1 ± 3.3	6.4 ± 4
*p*	—	—	<0.001	0.204	0.874	0.002	<0.001			<0.001	0.837				0.634	0.785	0.206

Demographic characteristics of the UK Biobank (subset with genetic information and caffeine intake) and the HypnoLaus (subset with genetic information, caffeine intake, and sleep variables) datasets. We compared moderate (≤3 cups/day) to high (≥4 cups/day) habitual caffeine intake. In the UK Biobank dataset, total sleep time relies on self-reported habitual sleep duration, whereas in the HypnoLaus dataset, total sleep time refers to home polysomnography-recorded sleep duration. The *p*-values refer to either Wilxocon or Chi-squared tests, depending on whether continuous or categorical variables were analyzed (the corresponding *t*-tests revealed similar results—see Supplemental Table S3). Diurnal preference was based on the Horne-Östberg Morningness-Eveningness Questionnaire (MEQ). Sleep quality = self-rated sleep quality based on Pittsburgh Sleep Quality Index (PSQI). Daytime sleepiness was measured based on the Epworth Sleepiness Scale (ESS). Whenever possible, the mean and standard deviation are given.

**Figure 2.**
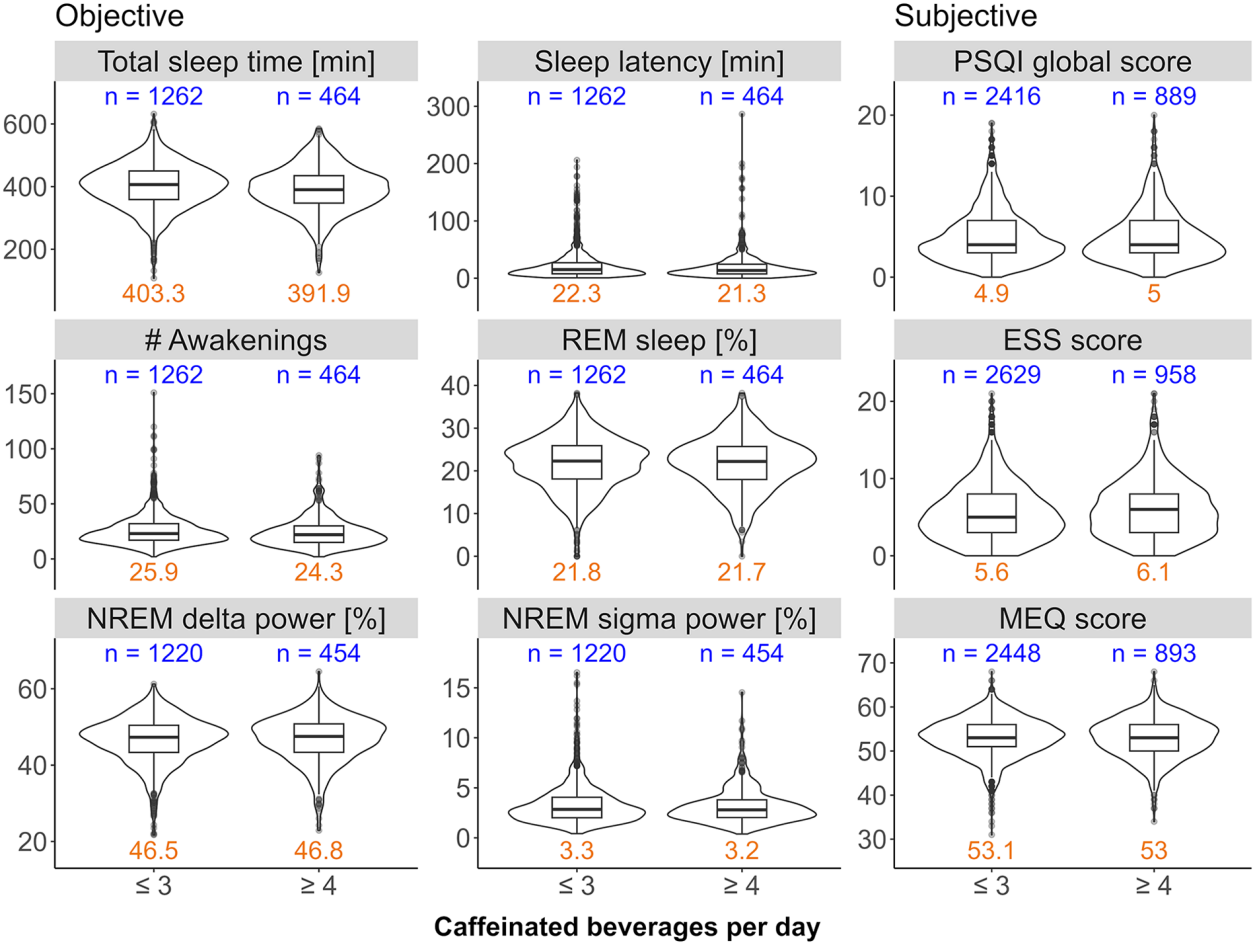
Distributions of sleep variables collected in the HypnoLaus dataset. The objective sleep variables in the HypnoLaus cohort for at most *n* = 1726 observations, the subjective variables for *n* = 3587 observations. The caffeine intake groups ⩽3 cups/day (moderate) and ⩾4 cups/day (high) are compared on the *x*-axis. The left and middle panels illustrate home polysomnography-derived, objective sleep quality: total sleep time (minutes), sleep latency (min, time between lights-out and first occurrence of stage N2 sleep), number (#) of awakenings, REM sleep (expressed as % of total sleep time), EEG delta power in NREM sleep (spectral power in the 1–4 Hz range expressed as a % of total power), and EEG sigma power in NREM sleep (spectral power in the 12–16 Hz range expressed as a % of total power). The right panel illustrates self-reported measures of sleep quality: Pittsburgh Sleep Quality Index (PSQI, global score), Epworth Sleepiness Scale (ESS) score, and Morningness-Eveningness Questionnaire (MEQ) score. *X*-axes: self-reported intake of caffeinated beverages per day. The blue values on top of each panel indicate the sample size per group. The orange values on the bottom of each panel indicate the mean value of the corresponding distribution.

The distributions of total sleep time, sleep latency, number of awakenings, percentage of REM sleep, proportions of delta (1–4 Hz) and sigma (12–16 Hz) power in the NREM sleep EEG, and the PSQI, EES and MEQ scores in moderate (⩽3 cups/day) and high (⩾4 cups/day) coffee consumers can be seen in Figure 2. The distributions of the more fine-grained caffeine intake levels can be found in the Supplemental Figure S4. The data show high variability in objective and subjective sleep quality measures as expected in a heterogeneous, community-derived study sample. Particularly, the variables sleep latency, number of awakenings, PSQI and ESS scores, and relative NREM sigma power display non-Gaussian distributions.

**Figure 3.**
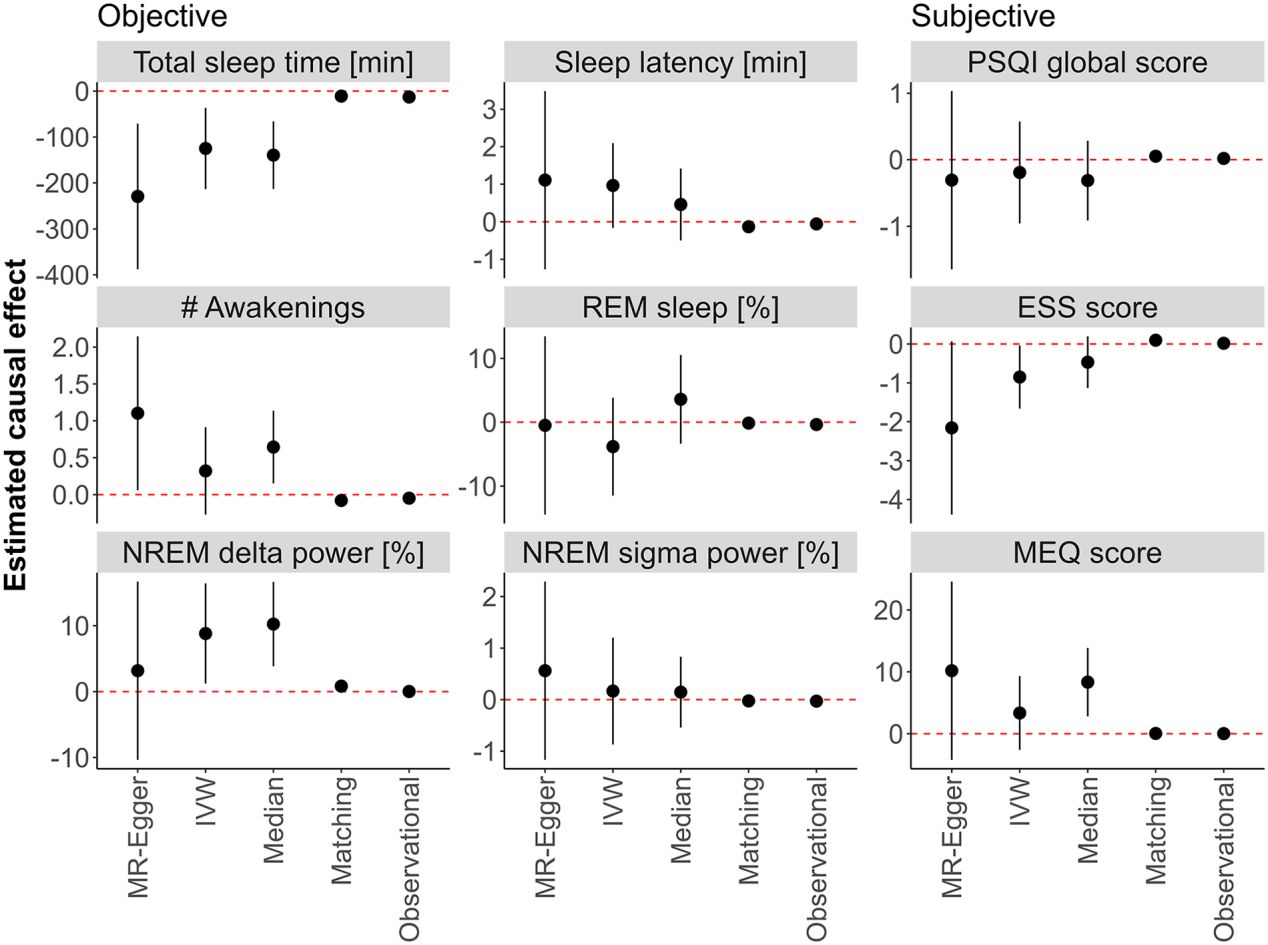
Mendelian randomization (MR), causal matching, and observational results. The effects estimated between participants in the HypnoLaus dataset (*n* = 1702) reporting high (⩾4 caffeinated beverages per day) and moderate (⩽3 caffeinated beverages per day) habitual caffeine intake is displayed. The left and middle panels illustrate home polysomnography-derived, objective sleep quality: total sleep time (minutes), sleep latency (min, time between lights-out and first occurrence of stage N2 sleep), number (#) of awakenings, REM sleep (expressed as % of total sleep time), EEG delta power in NREM sleep (spectral power in the 1–4 Hz range expressed as a % of total power), and EEG sigma power in NREM sleep (spectral power in the 12–16 Hz range expressed as a % of total power). The right panel illustrates self-reported measures of sleep quality: Pittsburgh Sleep Quality Index (PSQI, global score), Epworth Sleepiness Scale (ESS) score, and Morningness-Eveningness Questionnaire (MEQ) score. Estimators include the MR methods MR-Egger, inverse variance weighting (IVW) and weighted median (Median), the Matching Frontier algorithm (Matching), and the observational effect (Observational). The black dots indicate the estimated difference, and the dashed line represents no change. Error bars refer to the 95% confidence intervals. The values on the y-axes were log-transformed for sleep latency, number of awakenings, PSQI global score, and ESS score. The values of NREM sigma power are logit-transformed. Other values were not transformed.

All three MR methods (MR-Egger, IVW, and weighted median) revealed no differences between the groups in sleep latency, the percentage of REM sleep, sigma power in NREM sleep, nor self-rated sleep quality (PSQI global score; Figure 3). One or two, but not all three MR methods, suggested an effect of high caffeine intake on the number of awakenings (MR-Egger: estimate = 1.102, *p* = 0.038; weighted median: estimate = 0.64, *p* = 0.01), delta power in NREM sleep (IVW: estimate = 8.82%, *p* = 0.02; weighted median: estimate = 10.25%, *p* = 0.002), daytime sleepiness (ESS score; IVW: estimate = −0.85, *p* = 0.04), and diurnal preference (MEQ) (weighted median: estimate = 8.34, *p* = 0.003). When looking at total sleep time, all three methods showed shorter sleep in individuals reporting the consumption of ⩾4 caffeinated beverages per day (MR-Egger: estimate = −229 minutes, *p* = 0.03; IVW: estimate = −125 minutes, *p* = 0.005; weighted median: estimate = −140 minutes, *p* < 0.001; Figure 3).

After correction for multiple testing with the Holm-Bonferroni adjustment, the weighted median estimate for delta power in NREM sleep (*p* = 0.044) and total sleep time (*p* = 0.006) remained significant. All three MR-estimators exhibited high uncertainty, likely due to the inherently weak genetic instruments. This was particularly true for the MR-Egger estimator. Thus, the high mean difference (>2 hours) between the high and moderate caffeine consumers estimated with these methods appears unrealistically large.

The causal matching revealed no differences for the percentage of REM sleep, sigma power in the NREM sleep EEG, subjective sleep quality, and diurnal preference. By contrast, it showed shorter sleep latency (estimate = −0.1 minutes, *p* = 0.003), lower number of awakenings (estimate = −0.079, *p* = 0.003), and enhanced daytime sleepiness (estimate = 0.097, *p* < 0.001) in the high caffeine intake group. Corroborating the results of the MR, causal matching confirmed a shorter total sleep time per night (estimate = −11 minutes, *p* = 0.007) and higher delta power in the NREM sleep EEG (estimate = 0.8%, *p* = 0.018) in the high caffeine intake group. Although the effect sizes were rather small (total sleep time: Cohen’s *d* = −0.15; delta power: Cohen’s *d* = 0.14), the difference in total sleep time was strikingly similar to the observational effect (−12.9 minutes, *p* < 0.001; Figure 3).

For the other variables analyzed, the observational effects did not suggest a significant difference between the groups. As for the control outcome age, no causal model revealed a significant effect, whereas the observational effect was significant (*p* < 0.001, see Table S2). We report the complete results, including estimates, standard errors, confidence intervals, *p*-values, and Cohen’s *d* coefficients in Supplemental Table S2.

## Discussion

**Figure 4.**
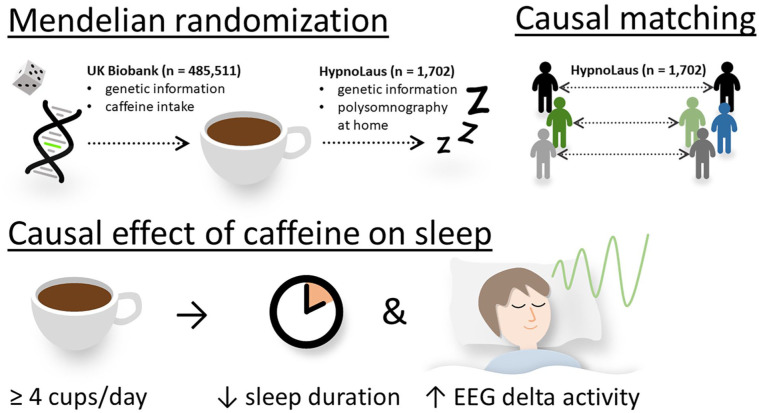
Summary of methods and datasets used and the results. Various two-sample Mendelian randomization techniques, including MR-Egger, inverse variance weighting, and weighted median, were employed to estimate the causal effect of habitual caffeine intake on objective and subjective sleep quality. Mendelian randomization makes use of the natural random allocation of single-nucleotide polymorphisms influencing caffeine intake, as estimated from the UK Biobank (*n* = 485,551). Tracking this genetic influence enables estimating causal effects of caffeine on both objective and subjective sleep variables, as captured in the HypnoLaus dataset (*n* = 1702). To further support this causal analysis, participants were matched across caffeine intake groups based on key characteristics of the HypnoLaus dataset, ensuring greater comparability with respect to known confounders. This causal matching was used to confirm the Mendelian randomization results. We consistently found that individuals consuming high levels of caffeine (⩾4 cups/day) exhibited reduced sleep duration, while relative EEG delta activity was elevated compared to those consuming ⩽3 cups/day. This suggests that habitual caffeine intake may trigger compensatory mechanisms in sleep and that the homeostatic regulation remains intact.

To study how habitual caffeine intake causally affects objective and subjective sleep quality, we applied three distinct MR methods, causal matching and observational effects, in two large datasets of community-based cohorts from the UK and Switzerland. We found consistent evidence for causally reduced total sleep time in individuals consuming ⩾4 caffeinated beverages per day compared to those consuming ⩽3 caffeinated beverages per day. While MR methods likely overestimated the difference due to moderate genetic associations, causal matching and observational estimates suggest a shortening of sleep by 11–13 minutes per night. By contrast, sleep depth as estimated by the percentage of EEG delta activity in NREM sleep was slightly enhanced in high compared to moderate habitual caffeine consumers. These main findings of the study are schematically summarized in Figure 4. Given the importance of adequate sleep for health (Luyster et al., 2012), even this small difference warrants careful consideration (Gallicchio and Kalesan, 2009).

The effects of chronic and repeated caffeine consumption on self-reported and objective sleep variables were investigated in a MR study and in randomized controlled trials. The first two-sample MR approach, however, was rather inconclusive (Treur et al., 2018). The authors sampled self-reported sleep duration with a resolution in hours, which weakens causal estimates. Self-reported sleep quality often differs from objective sleep and can vary across different populations (Bianchi et al., 2013; Rezaie et al., 2018). Consistent with our study, this work found no causal effect of caffeine on subjective sleep quality, suggesting that objective and subjective measures capture distinct caffeine effects on sleep quality.

Previous randomized studies also failed to find significant effects of two to three daily caffeine doses on total sleep time (Weibel et al., 2020, 2021; Zhang et al., 2020). However, these studies only involved 9 and 14 days of repeated caffeine administration in small samples (11 and 20 participants), studied under controlled conditions with strict bedtimes (Weibel et al., 2020, 2021; Zhang et al., 2020). Such settings do not realistically reflect the chronic caffeine intake and the common sleep behavior in the general population (Rochat et al., 2019).

Another randomized, cross-over trial estimated sleep duration across 14 days in 100 adults with a wrist-worn, commercial fitness tracker while alternating between consecutive 2-day periods with coffee or no coffee consumption (Marcus et al., 2023). Sleep duration was estimated to be 36 minutes shorter on coffee days compared to no-coffee days, which is roughly compatible with our findings. But changing between coffee and no-coffee days may cause carry-over or withdrawal effects, and wearables may not accurately measure sleep and have low accuracy in individual recordings (Stucky et al., 2021). Experiments in cats showed no effect of chronic caffeine intake on sleep duration (Sinton and Petitjean, 1989), which is in contrast to our results. However, the low sample size (*n* = 5) and the different species may preclude direct comparison to our findings in humans.

Controlled experiments in rodents and humans revealed EEG-derived evidence that repeated caffeine intake over several days can enhance sleep pressure when compared to placebo (Doty et al., 2017; Panagiotou et al., 2019; Weibel et al., 2020, 2021). In accordance with these findings, two different MR methods (IVW and median weighted) used in the present study suggested higher delta power in NREM sleep in the high caffeine group when compared to the moderate caffeine group. The median weighted method withstood correction for multiple testing. Although the MR-Egger estimate was not significant, the causal matching analysis confirmed increased delta power in NREM sleep. These findings may suggest that chronically reduced total sleep time from high caffeine consumption is compensated by enhanced sleep intensity. This regulatory principle is well known from controlled experiments (Åkerstedt et al., 2009). If confirmed in large studies, this would indicate that habitual caffeine use does not impact the homeostatic aspect of sleep-wake regulation, which is consistent with the notion that caffeine cannot compensate for lost sleep. It would be interesting to corroborate this with polysomnographic recordings in large and diverse participant samples, yet such measurements are time-consuming, expensive, and not readily available. While not as accurate, wearable sleep data could be a more practical option for gathering objective data as part of large genetic studies.

Several limitations apply to this work. First, the treatment variable (cups of caffeinated beverages per day) was self-reported and may be susceptible to recall bias (Laaboub et al., 2024). Additionally, we averaged over beverages with different caffeine contents, while potentially missing other dietary sources of caffeine (Drewnowski and Rehm, 2016). The large sample sizes can mitigate this concern. Indeed, the reported caffeine use in the two cohorts is similar to previous studies (Gracia-Lor et al., 2017; Rochat et al., 2019). Second, instrumental variable estimators typically suffer from low accuracy, especially if the instruments are weak. On the other hand, causal matching does not suffer from weak instruments but from potential unobserved confounding or reverse causality. To address the limits of each method, we combined different MR and matching methods, and found consistent evidence that high caffeine use reduces total sleep time and increases delta power.. Additionally, no causal model estimated a significant effect for the control outcome age, providing further evidence in favor of this approach. Third, the assumptions of different MR methods are difficult to verify. For example, gene variants can affect the outcome in another way than via the treatment. More specifically, many SNPs selected affect liver functions and therefore may have an effect on sleep quality that is not modulated by coffee consumption but rather metabolism. However, the intercepts in the MR-Egger regression analysis showed no evidence for such pleiotropy. In addition, our use of distinct methods, each making different assumptions, reduced the risk of violated assumptions skewing the overall results. Fourth, most participants in the two cohorts are of European descent. Large studies in more diverse populations are necessary to generalize the present results. Fifth, not all aspects of the sleep EEG, for example, gamma band activity, could be analyzed. Thus, we cannot rule out other effects of habitual caffeine intake on sleep microstructure. Finally, naps are not recorded in the HypnoLaus cohort. We cannot exclude that a reduced propensity to nap in the high caffeine intake group contributed to the observed increase in NREM delta power.

In conclusion, we used four complementary statistical methods in two large, high-quality, community-based datasets, to estimate the causal effects of chronic high caffeine intake on objective and subjective sleep quality estimates recorded at home. Compared to three or less caffeine containing beverages per day, we show that habitual consumption of more than four caffeinated drinks per day reduces total sleep time and increases sleep intensity as measured by EEG delta power in NREM sleep.

## Supplemental Material

sj-docx-1-jop-10.1177_02698811251368364 – Supplemental material for Community-based causal evidence that high habitual caffeine consumption alters distinct polysomnography-derived sleep variables Supplemental material, sj-docx-1-jop-10.1177_02698811251368364 for Community-based causal evidence that high habitual caffeine consumption alters distinct polysomnography-derived sleep variables by Benjamin Stucky, Leonard Henckel, Marloes H. Maathuis, José Haba-Rubio, Pedro Marques-Vidal, Francesca Siclari, Raphaël Heinzer and Hans-Peter Landolt in Journal of Psychopharmacology
