# Supplementary material for: Community-based causal evidence that high habitual caffeine consumption alters distinct polysomnography-derived sleep variables
Source: J Psychopharmacol. 2025 Oct 18;39(12):1437–48. doi: 10.1177/02698811251368364 (PMC12672940; doi:10.1177/02698811251368364)
Supplement: sj-docx-1-jop-10.1177_02698811251368364 – Supplemental material for Community-based causal evidence that high habitual caffeine consumption alters distinct polysomnography-derived sleep variables [file sj-docx-1-jop-10.1177_02698811251368364.docx]

**Community-based causal evidence that high habitual caffeine consumption alters distinct polysomnography-derived sleep variables**

Benjamin Stucky ^1,*^, Leonard Henckel ^2,*^, Marloes H. Maathuis, José Haba-Rubio ^3^, Pedro Marques-Vidal ^4^, Francesca Siclari ^3,5,6^, Raphaël Heinzer ^3,7^, Hans-Peter Landolt ^1,8^

*^1^ Institute of Pharmacology and Toxicology, University of Zurich, Zurich, Switzerland*

*^2^ School of Mathematics and Statistics, University College Dublin, Dublin, Ireland*

*^3^ Center for Investigation and Research in Sleep (CIRS), Lausanne University Hospital (CHUV), Lausanne, Switzerland*

*^4^ Department of Medicine, Internal Medicine, Lausanne University Hospital (CHUV) and University of Lausanne, Lausanne, Switzerland*

*^5^ The Netherlands Institute for Neuroscience, Amsterdam, the Netherlands*

*^6^ The Sense Innovation and Research Center, Lausanne and Sion, Switzerland*

*^7^ Pulmonary Department, Lausanne University Hospital (CHUV), Lausanne, Switzerland*

*^8^ Sleep & Health Zurich, University Center of Competence, University of Zurich, Zurich, Switzerland*

**Supplementary material**

**Address for correspondence**Benjamin Stucky, PhD
Institute of Pharmacology & Toxicology
University of Zürich
Winterthurerstrasse 190
8057 Zürich, Switzerland
Tel.: +41 44 635 59 55
e-mail: [benjamin.stucky@pharma.uzh.ch](mailto:landolt@pharma.uzh.ch)

Figure S1. Causal matching estimates per units dropped.
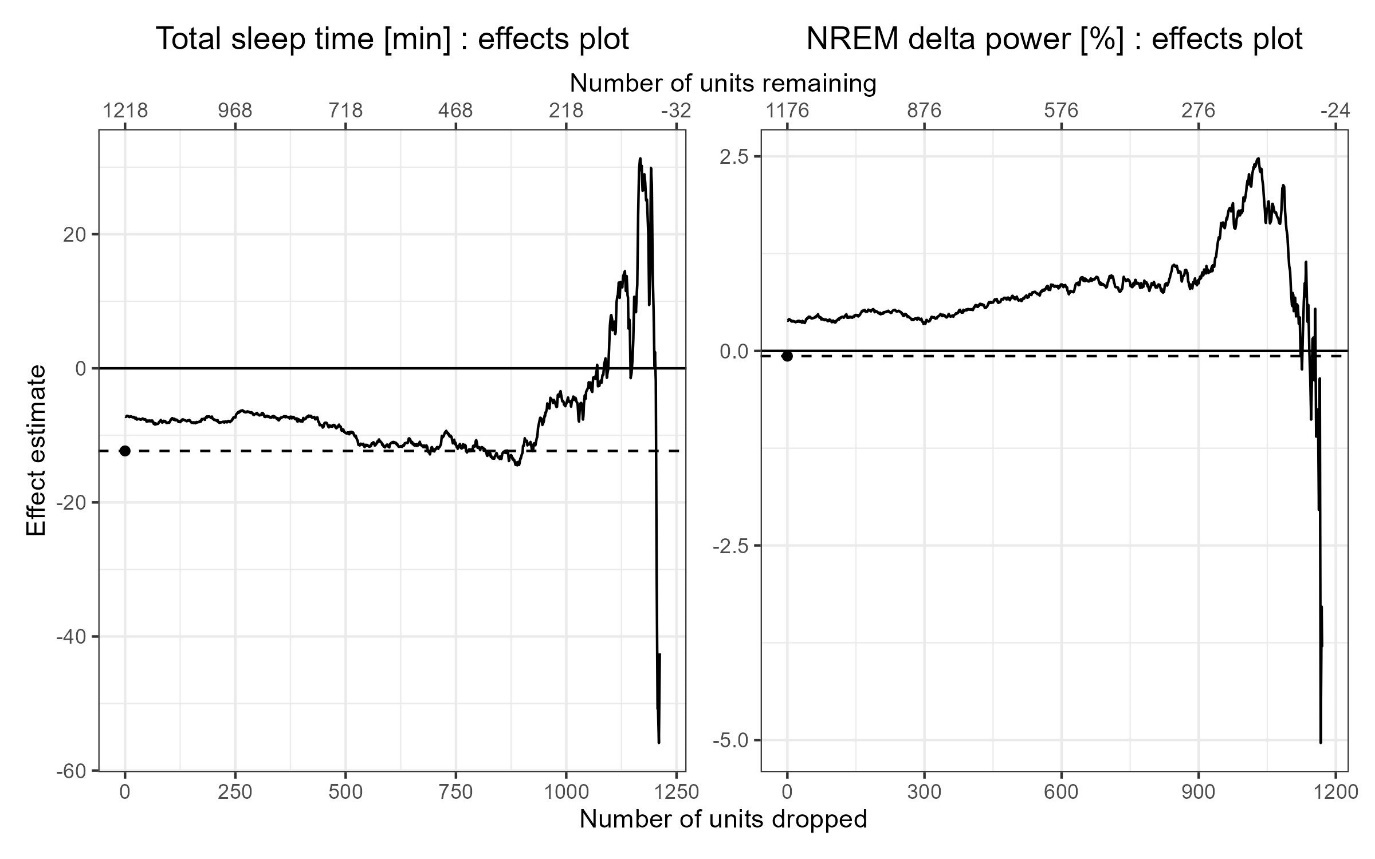


Shown are the matching estimates for the effect on total sleep time and NREM delta power as a percentage of total power per number of units dropped when using the matching from the MatchingFrontier R package.


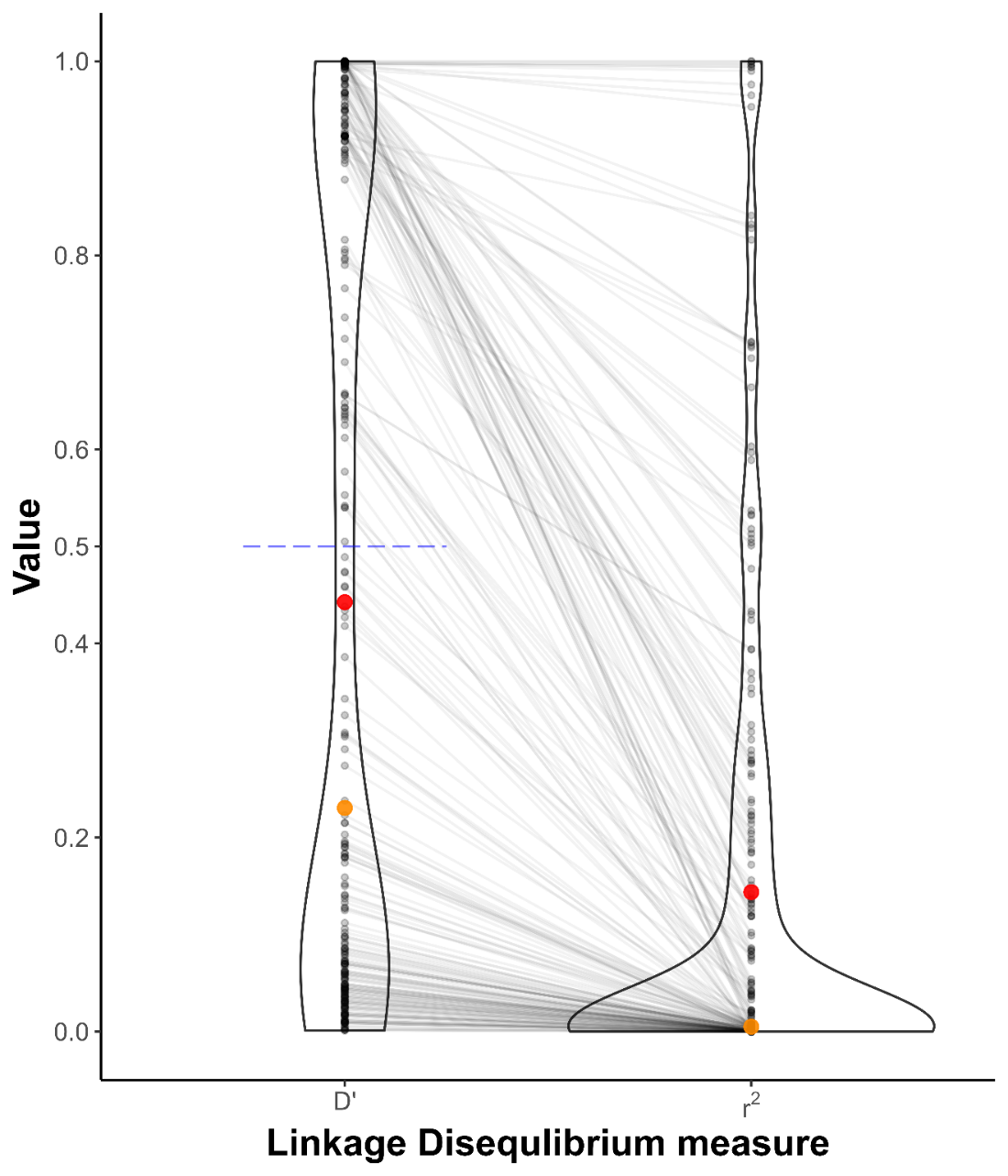
Figure S2. Comparing Linkage Disequilibrium measures.

The plot shows the distribution of two relative measures of Linkage Disequilibrium, D’ and r2, across all 83 selected SNPs. The red dots indicate the mean, the orange dots the median. The blue line displays the cut-off point 0.5 for D’. The grey lines between the two measures display how individual pairs of SNPs differ in their respective Linkage Disequilibrium measures. D’ exhibits a sharper separation of high to low Linkage Disequilibrium and captures non-linear linkage. The cut-off point 0.5 is the halfway point of the scale. Incidentally it is also close to the mean of the distribution.


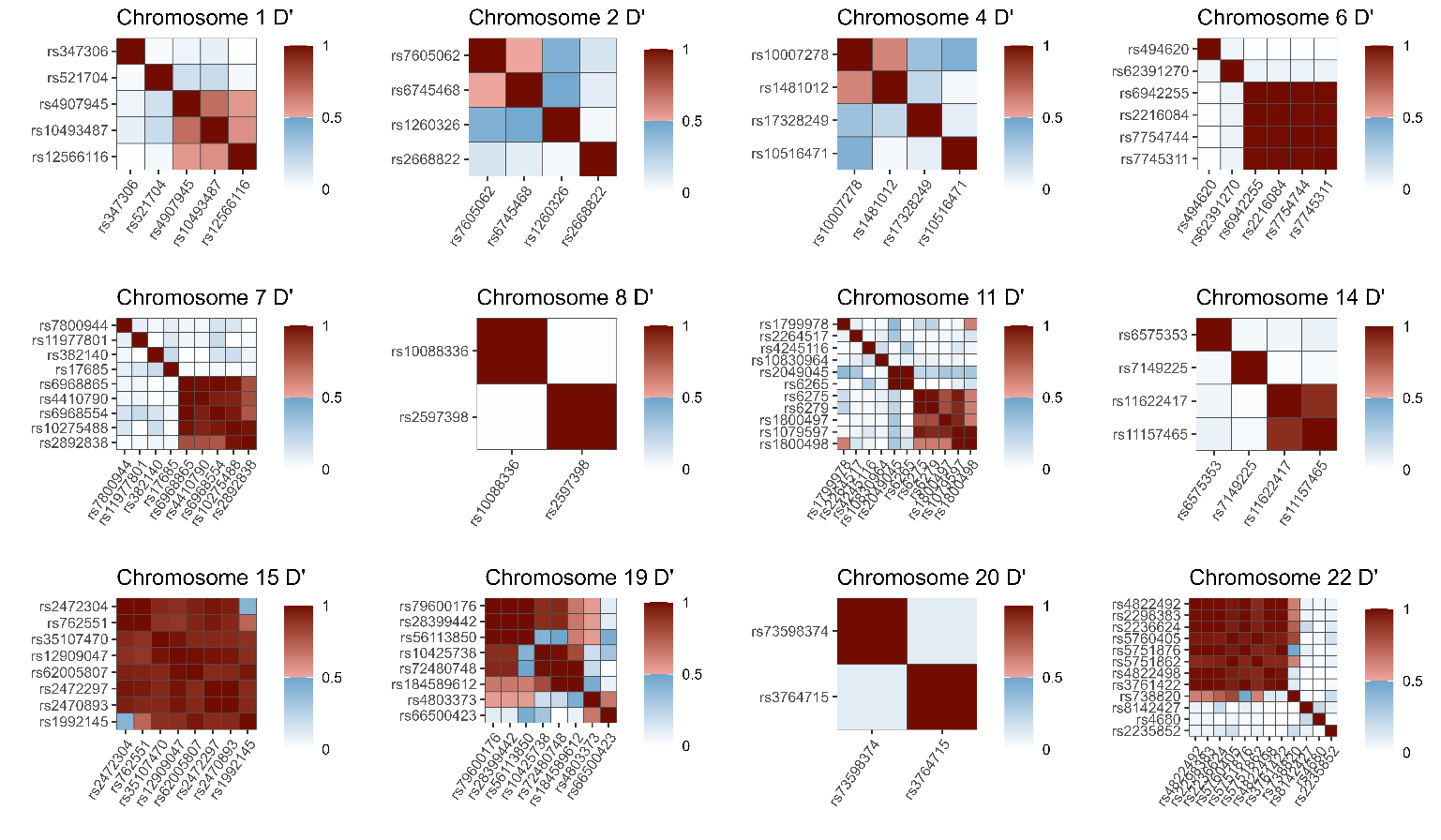
Figure S3. High linkage groupings.

Shown are the D‘ values between each SNP in a given Chromosome from the LDlink online tool. Values below the threshold of 0.5 in blue indicate weak linkage disequilibrium and values above 0.5 in red indicate high linkage disequilibrium. Variables are grouped by hierarchical clustering to show connected groupings.

**Figure S4.** Objective and subjective sleep variables in the HypnoLaus cohort.


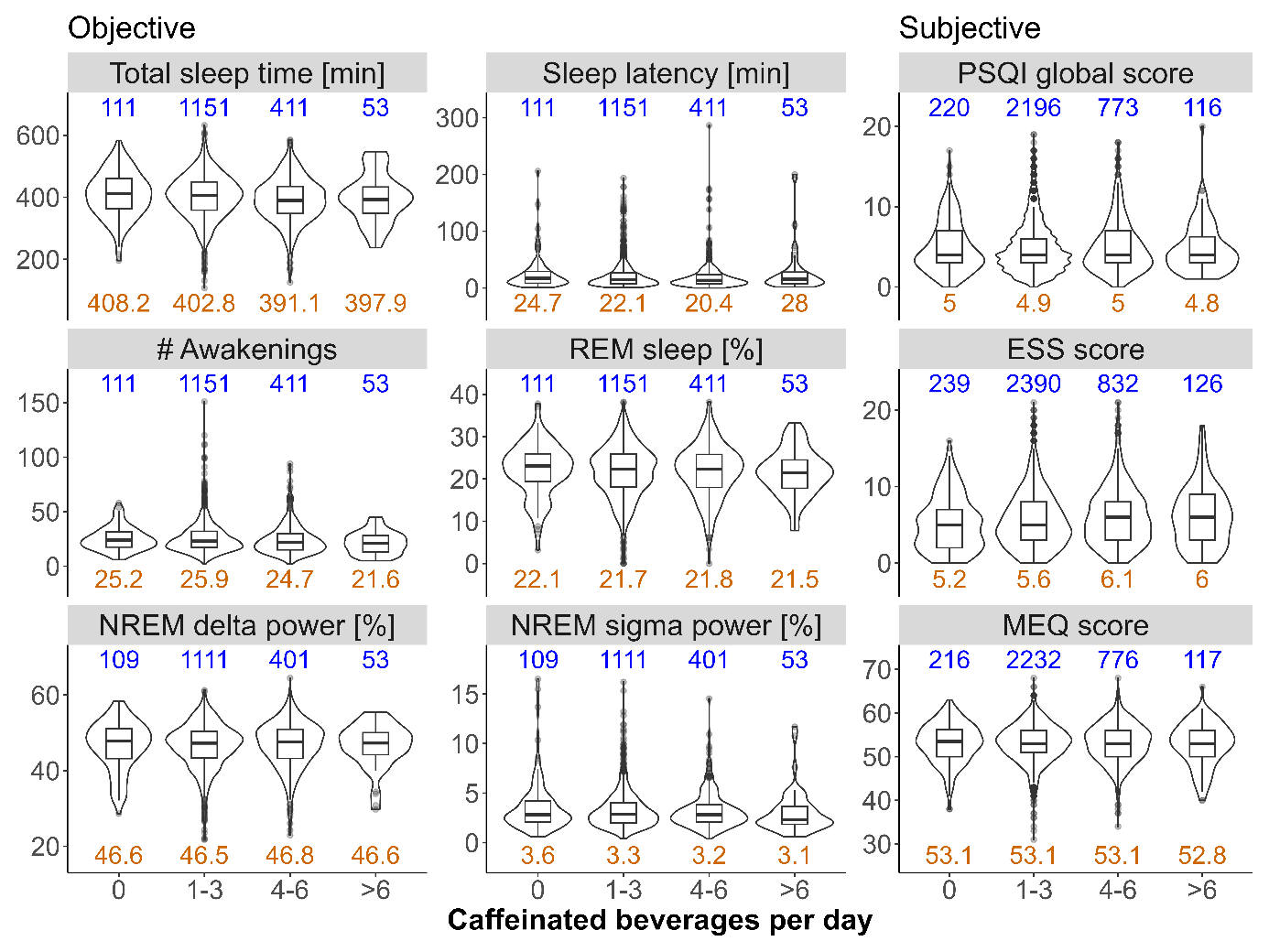


The caffeine intake groups 0 cups/day, 1-3 cups/day, 4-6 cups/day, >6 cups/day are compared on the x-axis. The left and middle panels illustrate home polysomnography-derived, objective sleep quality: total sleep time (min), sleep latency (min, time between lights-out and first occurrence of stage N2 sleep), number (#) of awakenings, REM sleep (expressed as % of total sleep time), EEG delta power in NREM sleep (spectral power in the 1-4 Hz range expressed as a % of total power), and EEG sigma power in NREM sleep (spectral power in the 12-16 Hz range expressed as a % of total power). The right panel illustrates self-reported measures of sleep quality: Pittsburgh Sleep Quality Index (PSQI, global score), Epworth Sleepiness Scale (ESS) score, and Morningness-Eveningness Questionnaire (MEQ) score. X-axes: self-reported intake of caffeinated beverages per day. The blue values on top of each panel indicate the sample size per group. The orange values on the bottom of each panel indicate the mean value of the corresponding distribution. For the subjective variables, at most n = 1,726 observations, and for the subjective variables, at most n = 3,587 observations were available.

Table S1. Demographic characteristics of HypnoLaus and UKBiobank cohorts.

|  | **Variable** | **Frequency** | **Overall** | **Moderate** | **High** | **High - Moderate** | **P (t)** | **P (wilcox)** | **P (χ2)** |
| --- | --- | --- | --- | --- | --- | --- | --- | --- | --- |
| **HypnoLaus** | Sample size |  | 1'726 | 1'262 | 464 | -798 | — | — | — |
|  | Age [y] |  | 58.54 (10.58) | 59.13 (10.63) | 56.94 (10.3) | -2.19 | <0.001 | <0.001 | — |
|  | Gender | Male | 840 | 602 | 238 | 3.6 % | — | — | 0.204 |
|  |  | Female | 886 | 660 | 226 | -3.6 % |  |  |  |
|  | BMI |  | 26.24 (4.41) | 26.23 (4.39) | 26.26 (4.48) | 0.03 | 0.902 | 0.874 | — |
|  | Total sleep time [h] |  | 6.67 (1.19) | 6.72 (1.18) | 6.53 (1.21) | -0.19 | 0.004 | 0.002 | — |
|  | Smoking status | Non-smoker | 721 | 576 | 145 | -14.4 % | — | — | <0.001 |
|  |  | Current | 314 | 190 | 124 | 11.7 % |  |  |  |
|  |  | Former | 688 | 494 | 194 | 2.7 % |  |  |  |
|  | Cigarettes per day |  | 14.91 (10.44) | 13.39 (10.03) | 17.18 (10.67) | 3.79 | 0.003 | <0.001 | — |
|  | Alcohol intake frequency | >2 / day | 28 | 21 | 7 | -0.2 % | — | — | 0.837 |
|  |  | 2 / day | 156 | 123 | 33 | -3.2 % |  |  |  |
|  |  | 1 / day | 230 | 183 | 47 | -5.2 % |  |  |  |
|  |  | 3 - 6 / week | 294 | 204 | 90 | 3.5 % |  |  |  |
|  |  | 1 - 2 / week | 475 | 332 | 143 | 4.8 % |  |  |  |
|  |  | Less frequent | 303 | 221 | 82 | -0.1 % |  |  |  |
|  |  | Never | 6 | 3 | 3 | 0.5 % |  |  |  |
|  | Horne-Oestberg Score |  | 53.23 (4.25) | 53.25 (4.21) | 53.2 (4.36) | -0.05 | 0.835 | 0.634 | — |
|  | PSQI Score |  | 5.08 (3.28) | 5.06 (3.25) | 5.12 (3.34) | 0.06 | 0.757 | 0.785 | — |
|  | Epwort Score |  | 6.11 (3.78) | 6.02 (3.71) | 6.37 (3.96) | 0.35 | 0.11 | 0.206 | — |
| **UKBiobank** | Sample size |  | 485'511 | 355'914 | 129'597 | -226'317 |  |  |  |
|  | Age [y] |  | 56.54 (8.09) | 56.59 (8.13) | 56.42 (7.98) | -0.17 | <0.001 | <0.001 | — |
|  | Gender | Male | 222173 | 157353 | 64820 | -5.8 % | — | — | <0.001 |
|  |  | Female | 263338 | 198561 | 64777 | 5.8 % |  |  |  |
|  | BMI |  | 27.42 (4.79) | 27.3 (4.78) | 27.74 (4.79) | 0.45 | <0.001 | <0.001 | — |
|  | Total sleep time [h] |  | 7.1 (1.3) | 7.12 (1.29) | 7.07 (1.24) | -0.05 | <0.001 | <0.001 | — |
|  | Smoking status | Non-smoker | 264750 | 201514 | 63236 | 7.8 % | — | — | 0.721 |
|  |  | Current | 50960 | 31770 | 19190 | -5.9 % |  |  |  |
|  |  | Former | 167969 | 121242 | 46727 | -2 % |  |  |  |
|  |  | Prefer not to answer | 1817 | 1376 | 441 | 0 % |  |  |  |
|  | Cigarettes per day |  | 15.37 (8.55) | 14.71 (8.49) | 16.35 (8.5) | 1.63 | <0.001 | <0.001 | — |
|  | Alcohol intake frequency | Daily or almost daily | 98957 | 70687 | 28270 | -2 % | — | — | 0.368 |
|  |  | 3 - 4 / week | 112257 | 81316 | 30941 | -1 % |  |  |  |
|  |  | 1 - 2 / week | 125272 | 92033 | 33239 | 0.2 % |  |  |  |
|  |  | 1 - 3 / month | 54043 | 39542 | 14501 | -0.1 % |  |  |  |
|  |  | Special occasions only | 55737 | 41888 | 13849 | 1.1 % |  |  |  |
|  |  | Never | 38825 | 30138 | 8687 | 1.8 % |  |  |  |
|  |  | Prefer not to answer | 405 | 298 | 107 | 0 % |  |  |  |
|  | Subjective sleeplessness | Usually | 136768 | 100528 | 36240 | 0.3 % | — | — | 0.6 |
|  |  | Sometimes | 231196 | 170368 | 60828 | 0.9 % |  |  |  |
|  |  | Never/rarely | 117097 | 84686 | 32411 | -1.2 % |  |  |  |
|  |  | Prefer not to answer | 435 | 320 | 115 | 0 % |  |  |  |

Shown are overall values, split into the moderate (≤ 3 cups of caffeinated beverage per day) and high (≥ 4 cups of caffeinated beverage per day) caffeine intake groups, the difference (if it is a numeric value, then the mean difference, if a factor a percentual change), and the p-values for the t-test, Wilcoxon test and Chi-squared test (whenever applicable) for a variety of demographic measures in both the HypnoLaus and the UK Biobank cohorts.

Table S2. Results of Mendelian Randomization methods, causal matching estimator, and observational linear regression estimator.

| **Variable** | **Panel** | **Method** | **Estimate** | | **SE** | **CI 2.5%** | **CI 97.5%** | **p-value** | **Cohen's d** |
| --- | --- | --- | --- | --- | --- | --- | --- | --- | --- |
| Log # awakenings | objective | IVW | | 0.321 | 0.302 | -0.271 | 0.912 | 0.288 |  |
| Log # awakenings |  | Matching | | -0.079 | 0.026 | -0.131 | -0.027 | 0.003 | -0.166 |
| Log # awakenings |  | Median | | 0.644 | 0.251 | 0.152 | 1.136 | 0.010 |  |
| Log # awakenings |  | MR Egger | | 1.102 | 0.532 | 0.059 | 2.146 | 0.038 |  |
| Log # awakenings |  | Observational | | -0.048 | 0.025 | -0.097 | 0.001 | 0.054 |  |
| Log sleep latency [min] |  | IVW | | 0.965 | 0.576 | -0.163 | 2.094 | 0.094 |  |
| Log sleep latency [min] |  | Matching | | -0.132 | 0.057 | -0.244 | -0.021 | 0.020 | -0.136 |
| Log sleep latency [min] |  | Median | | 0.461 | 0.488 | -0.496 | 1.417 | 0.345 |  |
| Log sleep latency [min] |  | MR Egger | | 1.109 | 1.212 | -1.267 | 3.484 | 0.360 |  |
| Log sleep latency [min] |  | Observational | | -0.056 | 0.048 | -0.151 | 0.038 | 0.244 |  |
| Logit NREM sigma [%] |  | IVW | | 0.166 | 0.529 | -0.870 | 1.202 | 0.753 |  |
| Logit NREM sigma [%] |  | Matching | | -0.024 | 0.031 | -0.086 | 0.037 | 0.440 | -0.046 |
| Logit NREM sigma [%] |  | Median | | 0.146 | 0.350 | -0.541 | 0.832 | 0.678 |  |
| Logit NREM sigma [%] |  | MR Egger | | 0.561 | 0.882 | -1.168 | 2.291 | 0.525 |  |
| Logit NREM sigma [%] |  | Observational | | -0.029 | 0.031 | -0.089 | 0.031 | 0.348 |  |
| Logit sleep efficiency [%] |  | IVW | | -0.385 | 0.493 | -1.351 | 0.580 | 0.434 |  |
| Logit sleep efficiency [%] |  | Matching | | 0.058 | 0.044 | -0.029 | 0.144 | 0.193 | 0.068 |
| Logit sleep efficiency [%] |  | Median | | -0.216 | 0.417 | -1.033 | 0.602 | 0.605 |  |
| Logit sleep efficiency [%] |  | MR Egger | | -0.727 | 0.879 | -2.449 | 0.996 | 0.408 |  |
| Logit sleep efficiency [%] |  | Observational | | 0.048 | 0.041 | -0.032 | 0.128 | 0.238 |  |
| NREM delta [%] |  | IVW | | 8.815 | 3.881 | 1.209 | 16.421 | 0.023 |  |
| NREM delta [%] |  | Matching | | 0.827 | 0.348 | 0.144 | 1.510 | 0.018 | 0.140 |
| NREM delta [%] |  | Median | | 10.250 | 3.265 | 3.850 | 16.649 | 0.002 |  |
| NREM delta [%] |  | MR Egger | | 3.172 | 6.900 | -10.351 | 16.695 | 0.646 |  |
| NREM delta [%] |  | Observational | | 0.019 | 0.329 | -0.627 | 0.665 | 0.954 |  |
| REM sleep [%] |  | IVW | | -3.820 | 3.902 | -11.469 | 3.828 | 0.328 |  |
| REM sleep [%] |  | Matching | | -0.150 | 0.331 | -0.800 | 0.500 | 0.651 | -0.025 |
| REM sleep [%] |  | Median | | 3.586 | 3.541 | -3.354 | 10.527 | 0.311 |  |
| REM sleep [%] |  | MR Egger | | -0.503 | 7.115 | -14.449 | 13.443 | 0.944 |  |
| REM sleep [%] |  | Observational | | -0.369 | 0.321 | -0.998 | 0.260 | 0.250 |  |
| Total sleep time [min] |  | IVW | | -124.980 | 45.077 | -213.330 | -36.631 | 0.006 |  |
| Total sleep time [min] |  | Matching | | -10.980 | 4.070 | -18.973 | -2.987 | 0.007 | -0.155 |
| Total sleep time [min] |  | Median | | -139.572 | 37.602 | -213.270 | -65.873 | 0.000 |  |
| Total sleep time [min] |  | MR Egger | | -229.358 | 80.860 | -387.841 | -70.875 | 0.005 |  |
| Total sleep time [min] |  | Observational | | -12.904 | 3.760 | -20.279 | -5.529 | 0.001 |  |
| Log ESS score | subjective | IVW | | -0.850 | 0.415 | -1.663 | -0.036 | 0.041 |  |
| Log ESS score |  | Matching | | 0.097 | 0.025 | 0.047 | 0.147 | 0.000 | 0.148 |
| Log ESS score |  | Median | | -0.468 | 0.339 | -1.133 | 0.197 | 0.167 |  |
| Log ESS score |  | MR Egger | | -2.159 | 1.137 | -4.387 | 0.069 | 0.057 |  |
| Log ESS score |  | Observational | | 0.018 | 0.035 | -0.050 | 0.086 | 0.595 |  |
| Log PSQI global score |  | IVW | | -0.193 | 0.391 | -0.958 | 0.573 | 0.622 |  |
| Log PSQI global score |  | Matching | | 0.052 | 0.026 | 0.000 | 0.104 | 0.051 | 0.077 |
| Log PSQI global score |  | Median | | -0.314 | 0.305 | -0.912 | 0.284 | 0.304 |  |
| Log PSQI global score |  | MR Egger | | -0.307 | 0.684 | -1.647 | 1.032 | 0.653 |  |
| Log PSQI global score |  | Observational | | 0.018 | 0.032 | -0.044 | 0.080 | 0.564 |  |
| MEQ score |  | IVW | | 3.355 | 3.040 | -2.604 | 9.314 | 0.270 |  |
| MEQ score |  | Matching | | 0.054 | 0.166 | -0.272 | 0.381 | 0.743 | 0.013 |
| MEQ score |  | Median | | 8.338 | 2.817 | 2.816 | 13.860 | 0.003 |  |
| MEQ score |  | MR Egger | | 10.183 | 7.343 | -4.210 | 24.576 | 0.166 |  |
| MEQ score |  | Observational | | 0.030 | 0.246 | -0.452 | 0.513 | 0.901 |  |
| Age [years] | Control variable | IVW | | -5.011 | 10.716 | -19.356 | 9.334 | 0.719 |  |
| Age [years] |  | Matching | | -0.300 | 0.413 | -1.111 | 0.512 | 0.469 | -0.028 |
| Age [years] |  | Median | | 8.643 | 6.627 | -4.347 | 21.632 | 0.192 |  |
| Age [years] |  | MR Egger | | -7.784 | 13.087 | -33.434 | 17.866 | 0.552 |  |
| Age [years] |  | Observational | | -2.278 | 0.608 | -3.470 | -1.086 | 0.000 |  |

Shown are the results for the Mendelian Randomization methods MR-Egger, Inverse variance weighting (IVW) and Median, the causal matching estimator (MatchingFrontier) and the observational linear regression estimator. The objective and subjective variable panels correspond to those in the main paper. Outcome variables include, total sleep time, log sleep latency in minutes, delta power in NREM, logit sigma power in NREM and REM sleep in percentages, log number of awakenings, log ESS score, log PSQI global score and the MEQ score. As control variable, we added ‘age’ (years), which is not casually affected by caffeine intake (i.e., higher caffeine intake does not change someone’s age). We provide the effect estimates with their standard errors (SE), the 95% confidence interval, the corresponding p-value and the Cohen’s d effect size for the matching estimator.

Table S3. Summary of pre-selected single nucleotide polymorphisms.

| **t** | **SNP-id** | **Gene(s)** | **Chr** | **IVW** | **Median** | **MR Egger** | **Linkage** | **Validity** | **Interactions** | **Publications Implausibility** |
| --- | --- | --- | --- | --- | --- | --- | --- | --- | --- | --- |
| 34.3 | rs2472297 | *CYP1A1, CYP1A2* | 15 |  | yes |  | 1 | implausible | Alcohol | [1], [2], [3] |
| 32.0 | rs2470893 | *CYP1A1, CYP1A2* | 15 | yes | yes | yes | 1 | plausible |  |  |
| 31.3 | rs35107470 | *AC012435.2, ARID3B* | 15 | yes | yes |  | 1 | plausible |  |  |
| 30.2 | rs4410790 | *AHR, AC073332.1* | 7 | yes | yes | yes | 2 | plausible |  |  |
| 30.1 | rs6968865 | *AHR, AC073332.1* | 7 | yes | yes |  | 2 | plausible |  |  |
| 29.7 | rs2472304 | *CYP1A2* | 15 | yes | yes |  | 1 | plausible |  |  |
| 29.0 | rs12909047 | *AC012435.2, AC012435.1, UBL7-AS1* | 15 | yes | yes |  | 1 | plausible |  |  |
| 26.0 | rs6968554 | *AHR,AC073332.1* | 7 | yes | yes |  | 2 | plausible |  |  |
| 20.6 | rs1992145 | *SEMA7A* | 15 | yes | yes |  | 1 | plausible |  |  |
| 19.7 | rs62005807 | *CLK3* | 15 | yes | yes |  | 1 | plausible |  |  |
| -19.2 | rs10275488 | *AHR, AC073332.1* | 7 | yes | yes |  | 2 | plausible |  |  |
| 18.5 | rs2892838 | *AHR, AC073332.1* | 7 | yes | yes |  | 2 | plausible |  |  |
| 16.0 | rs762551 | *CYP1A2* | 15 |  | yes |  | 1 | implausible | Smoking | [4], [5] |
| 13.9 | rs56113850 | *CYP2A6, AC008537.1* | 19 |  | yes | yes | 3 | implausible | Smoking | [6], [7], [8], [9] |
| 11.9 | rs4822492 | *ADORA2A-AS1* | 22 |  |  |  | 4 | implausible | Adenosine | [10] |
| 11.8 | rs7800944 | *MLXIPL* | 7 |  | yes | yes |  | rather implausible | Williams Beuren syndrome | [11] |
| 11.6 | rs2298383 | *ADORA2A* | 22 |  |  |  | 4 | implausible | Adenosine | [10] |
| 11.1 | rs17685 | *POR* | 7 | yes | yes | yes |  | plausible |  |  |
| 11.1 | rs10516471 | *PPP3CA* | 4 |  | yes | yes |  | rather implausible | Diabetes | [12] |
| -11.0 | rs7605062 | *POTEI* | 2 | yes | yes | yes | 5 | plausible |  |  |
| 10.1 | rs5751876 | *ADORA2A* | 22 |  |  |  | 4 | implausible | Adenosine | [10] |
| 9.9 | rs1800498 | *DRD2* | 11 |  | yes | yes | 6 | implausible | Dopamine | [13] |
| 9.4 | rs2668822 | *—* | 2 |  | yes | yes |  | unknown |  |  |
| 8.7 | rs767778 | *—* | 13 |  | yes | yes |  | unknown |  |  |
| -8.6 | rs10007278 | *ARHGEF38* | 4 | yes | yes | yes | 7 | plausible |  |  |
| 8.4 | rs6575353 | *PRIMA1* | 14 |  | yes | yes |  | rather implausible | Acetylcholin | [14], [15] |
| 8.3 | rs347306 | *NOS1AP* | 1 |  | yes | yes |  | rather implausible | Depression, Schizophrenia | [16] |
| 8.2 | rs6279 | *DRD2* | 11 |  | yes |  | 6 | implausible | Dopamine, Alcohol | [[13],](http://www.sciencedirect.com/science/article/pii/S108707920600102Xonlinelibrary.wiley.com/doi/10.1111/j.1369-1600.2012.00490.x) [17] |
| 8.1 | rs1571536 | *GADD45G* | 9 |  | yes | yes |  | rather implausible | Cellular stress and sleep | [18] |
| 8.1 | rs66500423 | *NUMBL* | 19 |  | yes | yes | 8 | rather implausible | Nicotine Dependence | [19] |
| 8.0 | rs6275 | *DRD2* | 11 |  |  |  | 6 |  |  |  |
| -7.8 | rs6745468 | *EMX1* | 2 |  |  |  | 5 |  |  |  |
| -7.4 | rs2270969 | *MCCC1* | 3 |  |  |  |  |  |  |  |
| -6.9 | rs7745311 | *PDSS2* | 6 |  |  |  | 9 |  |  |  |
| -6.9 | rs9386630 | *PDSS2* | 6 |  |  |  | 9 |  |  |  |
| -6.8 | rs7754744 | *PDSS2, RPS24P12* | 6 |  |  |  | 9 |  |  |  |
| -6.8 | rs6942255 | *PDSS2* | 6 |  |  |  | 9 |  |  |  |
| -6.8 | rs2216084 | *PDSS2* | 6 |  |  |  | 9 |  |  |  |
| 6.5 | rs5751862 | *SPECC1L* | 22 |  |  |  | 4 |  |  |  |
| 6.4 | rs17328249 | *HAND2-AS1* | 4 |  |  |  |  |  |  |  |
| -6.4 | rs494620 | *SLC44A4* | 6 |  |  |  |  |  |  |  |
| 6.3 | rs521704 | *near GBP4* | 1 |  |  |  |  |  |  |  |
| 5.5 | rs62391270 | *RNU7-133P, AL353152.1* | 6 |  |  |  |  |  |  |  |
| -5.4 | rs9902453 | *EFCAB5* | 17 |  |  |  |  |  |  |  |
| -5.2 | rs1481012 | *ABCG2* | 4 |  |  |  | 7 |  |  |  |
| -5.1 | rs2264517 | *—* | 11 |  |  |  |  |  |  |  |
| -4.6 | rs1799978 | *DRD2* | 11 |  |  |  | 6 |  |  |  |
| -4.6 | rs1800497 | *ANKK1* | 11 |  |  |  | 6 |  |  |  |
| 4.3 | rs4822498 | *ADORA2A-AS1* | 22 |  |  |  | 4 |  |  |  |
| -4.2 | rs12566116 | *—* | 1 |  |  |  | 10 |  |  |  |
| 4.1 | rs382140 | *NRCAM, LAMB4* | 7 |  |  |  |  |  |  |  |
| 3.6 | rs72480748 | *CYP2A7P2* | 19 |  |  |  | 3 |  |  |  |
| 3.5 | rs4803373 | *CYP2F2P, AC008537.1* | 19 |  |  |  | 8 |  |  |  |
| 3.4 | rs2049045 | *BDNF, BDNF-AS* | 11 |  |  |  | 11 |  |  |  |
| 3.1 | rs2597979 | *PRH1, TAS2R14, AC018630.2* | 12 |  |  |  |  |  |  |  |
| 2.6 | rs5760405 | *SPECC1L* | 22 |  |  |  | 4 |  |  |  |
| -2.6 | rs7149225 | *NRXN3* | 14 |  |  |  |  |  |  |  |
| -2.6 | rs184589612 | *CYP2A7P2, CYP2G1P* | 19 |  |  |  | 3 |  |  |  |
| 2.3 | rs10425738 | *CYP2A7P2, CYP2B7P* | 19 |  |  |  | 3 |  |  |  |
| 1.9 | rs3761422 | *ADORA2A* | 22 |  |  |  | 4 |  |  |  |
| -1.9 | rs1079597 | *DRD2* | 11 |  |  |  | 6 |  |  |  |
| -1.8 | rs4239278 | *—* | 18 |  |  |  |  |  |  |  |
| -1.6 | rs2236624 | *ADORA2A* | 22 |  |  |  | 4 |  |  |  |
| 1.6 | rs11977801 | *—* | 7 |  |  |  |  |  |  |  |
| 1.4 | rs4907945 | *—* | 1 |  |  |  | 10 |  |  |  |
| 1.4 | rs1260326 | *GCKR* | 2 |  |  |  |  |  |  |  |
| -1.3 | rs28399442 | *CYP2A6, AC008537.1* | 19 |  |  |  | 3 |  |  |  |
| -1.3 | rs79600176 | *CYP2A7, AC008537.1* | 19 |  |  |  | 3 |  |  |  |
| -1.2 | rs10493487 | *NEGR1* | 1 |  |  |  | 10 |  |  |  |
| -1.2 | rs6265 | *BDNF, BDNF-AS* | 11 |  |  |  | 11 |  |  |  |
| -0.9 | rs73598374 | *ADA* | 20 |  |  |  |  |  |  |  |
| -0.9 | rs11157465 | *—* | 14 |  |  |  | 12 |  |  |  |
| -0.8 | rs2597398 | *LOC105379315* | 8 |  |  |  |  |  |  |  |
| 0.8 | rs11622417 | *LINC00871* | 14 |  |  |  | 12 |  |  |  |
| 0.7 | rs10830964 | *MTNR1B, RPL26P31* | 11 |  |  |  |  |  |  |  |
| 0.6 | rs10088336 | *—* | 8 |  |  |  |  |  |  |  |
| -0.2 | rs4245116 | *OPCML* | 11 |  |  |  |  |  |  |  |
| — | rs11863088 | *ATF7IP2* | 16 |  |  |  |  |  |  |  |
| — | rs2235852 | *RANGAP1* | 22 |  |  |  |  |  |  |  |
| — | rs3764715 | *SNPH* | 20 |  |  |  |  |  |  |  |
| — | rs4680 | *COMT* | 22 |  |  |  |  |  |  |  |
| — | rs738820 | *GUCD1* | 22 |  |  |  | 4 |  |  |  |
| — | rs8142427 | *FAM19A5* | 22 |  |  |  |  |  |  |  |

The table provides an overview of the 83 preselected SNPs and our decision regarding which to use for each MR method. The first row contains the t-values of the linear SNP-treatment regression sorted by absolute value. The high-linkage groups (D’ > 0.5) are color coded in the linkage column. Relevant publications regarding the implausibility of a SNPs validity are included for the first 30 SNPs, i.e., those SNPs that fulfill the inclusion criteria of having an absolute t-value larger than 8.

**References to Table S3**

1. Zhou H, Sealock JM, Sanchez-Roige S, Clarke T-K, Levey DF, Cheng Z, et al. Genome-wide meta-analysis of problematic alcohol use in 435,563 individuals yields insights into biology and relationships with other traits. Nat Neurosci. 2020;23:809–818.

2. Liu M, Jiang Y, Wedow R, Li Y, Brazel DM, Chen F, et al. Association studies of up to 1.2 million individuals yield new insights into the genetic etiology of tobacco and alcohol use. Nat Genet. 2019;51:237–244.

3. Zhong VW, Kuang A, Danning RD, Kraft P, van Dam RM, Chasman DI, et al. A genome-wide association study of bitter and sweet beverage consumption. Hum Mol Genet. 2019;28:2449–2457.

4. Wang L, Hu Z, Deng X, Wang Y, Zhang Z, Cheng Z-N. Association between Common CYP1A2 Polymorphisms and Theophylline Metabolism in Non-smoking Healthy Volunteers. Basic Clin Pharmacol Toxicol. 2013;112:257–263.

5. Sachse C, Brockmöller J, Bauer S, Roots I. Functional significance of a C→A polymorphism in intron 1 of the cytochrome P450 CYP1A2 gene tested with caffeine. Br J Clin Pharmacol. 1999;47:445–449.

6. Buchwald J, Chenoweth MJ, Palviainen T, Zhu G, Benner C, Gordon S, et al. Genome-wide association meta-analysis of nicotine metabolism and cigarette consumption measures in smokers of European descent. Mol Psychiatry. 2021;26:2212–2223.

7. Loukola A, Buchwald J, Gupta R, Palviainen T, Hällfors J, Tikkanen E, et al. A Genome-Wide Association Study of a Biomarker of Nicotine Metabolism. PLOS Genet. 2015;11:e1005498.

8. Patel YM, Park SL, Han Y, Wilkens LR, Bickeböller H, Rosenberger A, et al. Novel Association of Genetic Markers Affecting CYP2A6 Activity and Lung Cancer Risk. Cancer Res. 2016;76:5768–5776.

9. McKay JD, Hung RJ, Han Y, Zong X, Carreras-Torres R, Christiani DC, et al. Large-scale association analysis identifies new lung cancer susceptibility loci and heterogeneity in genetic susceptibility across histological subtypes. Nat Genet. 2017;49:1126–1132.

10. Erblang M, Drogou C, Gomez-Merino D, Metlaine A, Boland A, Deleuze JF, et al. The Impact of Genetic Variations in ADORA2A in the Association between Caffeine Consumption and Sleep. Genes. 2019;10:1021.

11. Goldman SE, Malow BA, Newman KD, Roof E, Dykens EM. Sleep patterns and daytime sleepiness in adolescents and young adults with Williams syndrome. J Intellect Disabil Res. 2009;53:182–188.

12. Meigs JB, Manning AK, Fox CS, Florez JC, Liu C, Cupples LA, et al. Genome-wide association with diabetes-related traits in the Framingham Heart Study. BMC Med Genet. 2007;8:1–10.

13. Monti JM, Monti D. The involvement of dopamine in the modulation of sleep and waking. Sleep Med Rev. 2007;11:113–133.

14. Hildebrand MS, Tankard R, Gazina EV, Damiano JA, Lawrence KM, Dahl H-HM, et al. PRIMA1 mutation: a new cause of nocturnal frontal lobe epilepsy. Ann Clin Transl Neurol. 2015;2:821–830.

15. Watson CJ, Baghdoyan HA, Lydic R. Neuropharmacology of Sleep and Wakefulness. Sleep Med Clin. 2010;5:513–528.

16. Cheah S-Y, Lawford BR, Young RMcD, Morris CP, Voisey J. Association of NOS1AP variants and depression phenotypes in schizophrenia. J Affect Disord. 2015;188:263–269.

17. Meyers JL, Nyman E, Loukola A, Rose RJ, Kaprio J, Dick DM. The association between DRD2/ANKK1 and genetically informed measures of alcohol use and problems. Addict Biol. 2013;18:523–536.

18. Naidoo N. Cellular stress/the unfolded protein response: Relevance to sleep and sleep disorders. Sleep Med Rev. 2009;13:195–204.

19. Hatoum AS, Colbert SMC, Johnson EC, Huggett SB, Deak JD, Pathak GA, et al. Multivariate genome-wide association meta-analysis of over 1 million subjects identifies loci underlying multiple substance use disorders. Nat Ment Health. 2023;1:210–223.
